# Effectiveness of an Intervention to Enhance Occupational Physicians’ Guideline Adherence on Sickness Absence Duration in Workers with Common Mental Disorders: A Cluster-Randomized Controlled Trial

**DOI:** 10.1007/s10926-016-9682-x

**Published:** 2016-11-30

**Authors:** Karlijn M. van Beurden, Evelien P. M. Brouwers, Margot C. W. Joosen, Michiel R. de Boer, Jaap van Weeghel, Berend Terluin, Jac J. L. van der Klink

**Affiliations:** 1 Department of Social and Behavioral Sciences, Tranzo Scientific Center for Care and Welfare, Tilburg University, PO Box 90153, 5000 LE Tilburg, The Netherlands; 2 Department of Health Sciences, Faculty of Earth and Life Sciences, VU University Amsterdam, De Boelelaan 1085, 1081 HV Amsterdam, The Netherlands; 3 Phrenos Centre of Expertise, PO Box 1203, 3500 BE Utrecht, The Netherlands; 4 Parnassia Group, Dijk en Duin Mental Health Center, PO Box 305, 1900 AH Castricum, The Netherlands; 5 Department of General Practice and Elderly Care Medicine, EMGO Institute for Health and Care Research, VU University Medical Center Amsterdam, PO Box 7057, 1007 MB Amsterdam, The Netherlands; 6 Netherlands School of Public & Occupational Health, PO Box 20022, 3502 LA Utrecht, The Netherlands

**Keywords:** Mental health, Occupational health service, Occupational medicine, Practice guideline, Return to work

## Abstract

*Purpose* Evidence-based guidelines in occupational health care improve the quality of care and may reduce sickness absence duration. Notwithstanding that, guideline adherence of occupational physicians (OPs) is limited. Based on the literature on guideline implementation, an intervention was developed that was shown to effectively improve self-reported adherence in OPs. The aim of present study was to evaluate whether this intervention leads to earlier return to work (RTW) in workers with common mental disorders (CMD). *Methods* In a two-armed cluster randomized controlled trial, 66 OPs were randomized. The trial included 3379 workers, with 1493 in the intervention group and 1886 in the control group. The outcome measures were: time to full RTW, time to first RTW, and total hours of sickness absence. Cox regression analyses and generalized linear mixed model analyses were used for the evaluations. *Results* The median time to RTW was 154 days among the 3228 workers with CMD. No significant differences occurred in (time to) full RTW between intervention and control group HR 0.96 (95% CI 0.81–1.15) nor for first RTW HR 0.96 (95% CI 0.80–1.15). The mean total hours of sickness absence was 478 h in the intervention group and 483 h in the control group. *Conclusions* The intervention to enhance OPs’ guideline adherence did not lead to earlier RTW in workers with CMD guided by the OPs. Possible explanations are the remaining external barriers for guideline use, and that perceived guideline adherence might not represent actual guideline adherence and improved care.

Trail registration: ISRCTN86605310.

## Introduction

As in many Western countries, in the Netherlands, sickness absence due to common mental disorders (CMD) is a problem that is associated with individual suffering and high costs for employers and society [1–3]. To improve the quality of occupational care, the Netherlands Society of Occupational Medicine (NVAB) developed (2000) and revised (2007) an evidence-based practice guideline named “Management of mental health problems of workers by occupational physicians” [4, 5]. Several studies have since been conducted on the effect of interventions aiming to improve the use of this guideline by occupational physicians (OPs) on workers outcomes. The first study, by van der Klink et al. [6] showed positive effects on the time to return to work (RTW); in this study, the occupational physicians were compliant with the intervention. In a retrospective study, researchers found that closer adherence to this guideline was associated with shortened sickness absence in workers with adjustment disorders [7]. In addition, Rebergen et al. found that OPs actual adherence to the guideline was limited, despite the fact that they had a positive attitude about using this guideline [8–10]. Apparently, implementing this guideline in practice is still challenging.

To improve adherence to this guideline, we developed a tailored implementation strategy based on findings from scientific implementation literature on how to improve guideline adherence [11–15]. According to the literature, more active implementation strategies are needed [12, 13] rather than dissemination among professionals and short introductions. Preferably, these active implementation strategies are tailored for a specific target group and setting, and they intend to eliminate perceived barriers that hinder physicians from using guidelines [11, 14, 16]. Moreover, to successfully overcome barriers for guideline use, the target users of a guideline should be actively involved in identifying barriers for specific guideline recommendations and selecting solutions [15]. In line with this aim, we developed an intervention to enhance OPs’ guideline adherence, focusing on identifying and solving the barriers for applying this guideline’s key recommendations. This intervention showed to be feasible in practice and effective in enhancing OPs’ knowledge, attitudes, perceived skills, and perceived guideline adherence; however, their perceived external barriers remained [17].

In the present cluster randomized controlled trial (RCT), we evaluated the tailored intervention to see whether it led to earlier and sustained RTW in workers who were sick-listed due to CMD compared to those receiving usual care. Specifically, we formulated the following research questions: What is the effect of the intervention aimed to enhance OPs’ guideline adherence on (1) the time to full RTW, (2) the time to first RTW in workers sick-listed due to CMD, (3) the total hours of sickness absence during a 1 year period after the start of the sickness absence?

## Methods

In the present paper, the “CONSORT 2010 statement: extension to cluster randomized controlled trials” [18] was used for reporting. A detailed description of the study protocol [19] and the intervention for OPs have been reported elsewhere [17].

### Study Context

According to the Dutch Gatekeeper Improvement Act, in case of sickness absence, both employer and worker are responsible for the recovery and return to work of the sick listed worker [20]. The employer is obliged to pay at least 70% of the wage during the sickness absence of a worker for a period of 2 years and to provide occupational health care. Sick listed workers have to consult an OP for diagnosis, assessment of the workability, and guidance within the first 6 weeks of the recovery and return to work process [20]. The OP has to manage this process with workers and their employer and supervisor.

### Trial Design

**Fig. 1:**
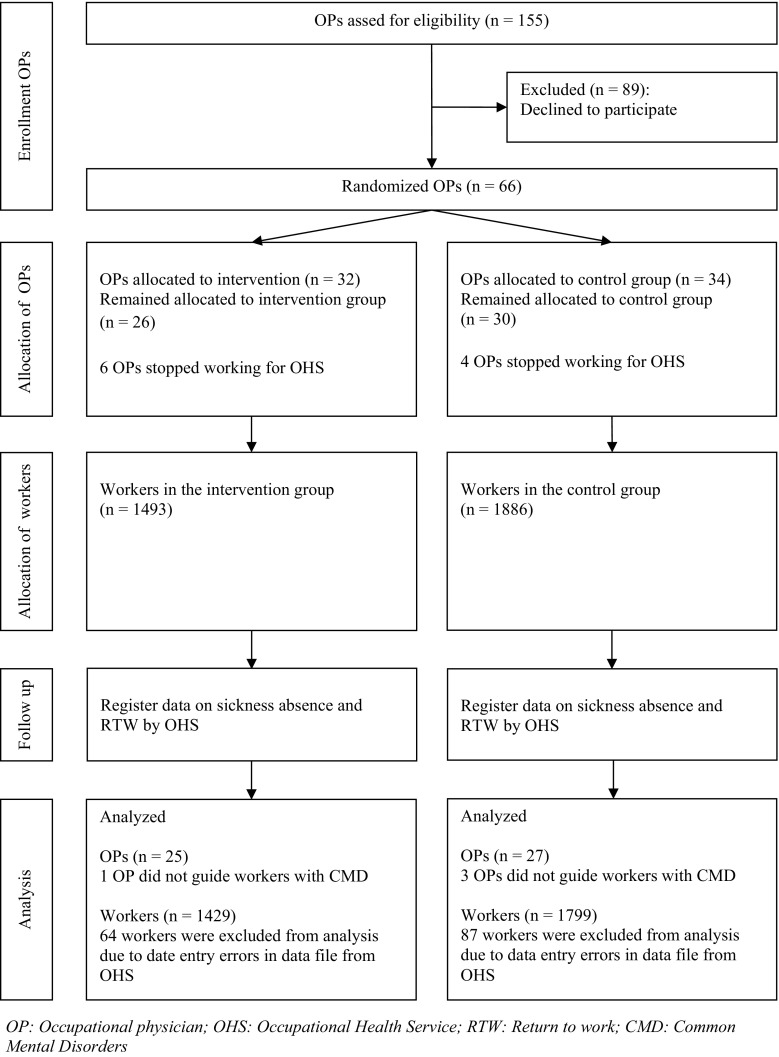
Flow diagram of this study

This study was designed as a two-armed cluster RCT with randomization at the level of the OP (Fig. 1).

The OPs were randomly allocated to the intervention group or to the control group. After completion of the 1 year intervention for the OPs, the registration of data on sickness absences and workers’ RTW was started from January 1st, 2012 until February 28th, 2014. The data were routinely recorded by the occupational health service (OHS) in their registration system, and for this study the data were extracted by the OHS from their registration system. The data provided to us were not traceable to the individual workers.

We obtained approval from the Medical Research Ethics Committee of St. Elisabeth Hospital in Tilburg, The Netherlands. This study was registered in the ISTCTN trial register, ISRCTN86605310.

### Participants

#### Occupational physicians

OPs were recruited between October 2010 and January 2011 from sites of a large OHS in the Netherlands. All 155 OPs of the sites in the Southern part of The Netherlands received written and oral information about the study. The 66 OPs participated on a voluntary basis and signed informed consents. After completing the intervention, the OPs in the intervention group received educational credits.

#### Workers

Eligible workers were between 18 and 64 years old, and had a first period of sickness absence between January 1st, 2012 and January 15th, 2013. All workers were receiving guidance by an OP who participated in the study and who had diagnosed the worker as having CMD (according to the Dutch Classification of Diseases, based on the ICD-10) [21]. The companies that workers were employed at, varied in size and served different sectors.

### Intervention

#### Intervention Group

Workers in the intervention group received guidance from an OP who had received the intervention to enhance OPs’ guideline adherence. A detailed description of this intervention has been published elsewhere [17]. In short, this intervention consists of an eight-session training in small peer-learning groups, takes place over 12 months, and is focused on barriers that hindered OPs from using specific recommendations in this guideline in practice. According to the model of Cabana et al. [22], guideline adherence can be affected by three main clusters of barriers: (1) lack of knowledge, (2) negative attitudes, and (3) external barriers. The OPs exchanged ideas and solutions to overcome the perceived barriers, drew up joint action plans on how to implement these solutions in their daily practice, and tested the suggested solutions in daily practice [17, 19].

Regarding the guideline content, the overall role of the OP is to monitor the process of sickness absence and RTW, to facilitate communication between workers and their employer and supervisor, to provide information and advice to the employer, supervisor, human resource management, and co-workers on how to support the worker and enhance his or her recovery and RTW, and to intervene in case of stagnation, either by OPs’ own interventions or by referral to a mental health specialist. According to the guideline, the guidance of a worker who is sick-listed with CMD starts with a problem orientation and an OP’s diagnosis. Next, the OP evaluates the worker’s recovery and RTW process by monitoring and enhancing the worker’s problem solving capacity according to the three phase model of Meichenbaum [23]. If the recovery process stagnates, the OP uses cognitive behavioral techniques to enhance the worker’s problem-solving capacity. Consultations with the worker take place every 3 weeks during the first 3 months, and then every 6 weeks thereafter. The OP contacts the supervisor or employer once a month [4, 24]. A detailed description of the content of the guideline has been reported elsewhere [17, 19, 25].

#### Control Group

As the guideline was distributed among Dutch OPs and became part of their medical education, guideline-based care came to be seen as usual care. However, subsequent research has shown that actual adherence to this guideline was limited [7–10]; therefore, in this study, care as usual was the guidance received by workers in the control group.

### Outcomes

The focus of the present study was on outcomes at the level of the workers. Workers’ personal baseline characteristics (age, gender, number of contract working hours per week), and data on sickness absence and RTW were extracted from the OHS registration system.

#### Primary Outcome

The time to the CMD workers’ full RTW was calculated as the number of calendar days between the first day of sickness absence and the first day of full RTW. Working the number of hours of their employment contract, for at least 4 weeks was considered a full RTW. The calculated time until full RTW was based on the data extracted from the OHS registration system.

#### Secondary Outcomes

Two secondary outcomes were assessed, i.e. time to first RTW and the total number of sick-leave hours. The time to the first RTW was calculated as the number of calendar days between the first day of sickness absence and the first day of RTW, irrespective of the number of working hours resumed in a week and the duration of this period. The total number of sick leave hours was calculated over a 1-year period, taking into account the total hours of their employment contract and partial RTW.

### Sample Size

We performed a power analysis to determine the sample size needed to detect a difference between the control and the intervention group with respect to the time to the CMD workers’ full RTW (primary outcome) and calculated the need to include a total of 232 workers (A detailed description of the performed sample size was published elsewhere [19]). Despite considerable efforts the recruitment of a representative group of workers was difficult for several reasons, e.g. employers gave no permission to invite their workers for the study or eligible workers were too tired to want to participate. Because recruitment resulted in too small a sample size, we subsequently used the anonymized sickness absence and RTW data of all 3379 workers sick listed due to CMD, who were guided by participating OPs during the study period. These data had already been recorded in the OHS registration system. This way, resulted in an unbiased and much larger data set. A consequence of using the anonymized data of 3379 workers was the limited number of available baseline characteristics, such as diagnosis, severity of CMD, aspects related to the work context, and treatment by other (mental) health care professionals that preferably would have been taken into account as possible confounders or effect modifiers in the analyses. The data of the 128 recruited workers will be used in other evaluations, separate from the current paper.

### Randomization

After recruitment, OPs were randomized by computerized allocation to the intervention or to the control group. The allocation was communicated to the OPs after the randomization of all participating OPs. Workers were allocated to the same group as their OP.

### Blinding

Workers and their companies were blinded for randomization since they were not aware of the allocation of their OP. The data collector who extracted the data from the registration system at the OHS and the researcher who assessed the survival outcomes (MdB) were also blinded for allocation of the OPs and of the workers to the intervention or to the control group. OPs were not informed about the inclusion of the workers they guided.

### Statistical Methods

#### Time Until Full RTW and Time Until First RTW

To evaluate the effect of the intervention, we performed intention-to-treat analyses. To illustrate the differences between the intervention and control group, we generated Kaplan–Meier survival curves, but for practical reasons, did not account for the multilevel design. Cox regression analysis was used to compare the difference between the intervention and the control group on the (time until) full and first RTW. To correct for the cluster design, we used the frailty random effect in this analysis [26]. Cox regression models the logarithm of the incidence or hazard rate, the number of new ‘events’ (i.e. RTW) per population ‘at-risk’ (i.e. sick-listed workers) per unit time. Workers were censored when the full RTW or the first RTW was not established within the follow up period (from the first day of sickness absence until February 28th 2014), or when the worker was lost to follow up within that period. The influence of baseline characteristics was evaluated using gender, age, and number of working hours as covariates in the model.

#### Total Hours of Sickness Absence

To evaluate the total hours of workers’ sickness absence, we used generalized linear mixed models analysis with inverse Gaussian distribution. The total hours of workers’ sickness absence was the dependent variable. Group (intervention of control group) was added as a fixed factor to the model.

Analyses were performed with R statistical program version 3.0.1 with the frailtypack [26] and SPSS version 19.0 (IBM Corp., Armonk, NY, 2010).

## Results

### Participant Flow and Baseline Data

A total of 66 OPs participated. As can be seen in Fig. 1, data of 3228 workers were analyzed, of which 280 workers did not establish full RTW and 214 workers did not establish first RTW within the follow up period. The mean follow up time was 595 days (SD 118) from first day of sick leave until February 28th 2014. Both groups contained more female than male workers. The number of contract working hours per week was comparable between both groups. See Table 1.

**Table 1:** Baseline characteristics of the participants per group

	Intervention group			Control group		
	Mean	SD	%	Mean	SD	%
Worker characteristic	(n = 1429)			(n = 1799)		
Gender, male	–	–	39.5	–	–	43.3
Age	45.1	11.1	–	44.1	10.8	–
Number of contract working hours per week	29.8	10.7	–	30.6	10.3	–
Occupation physician characteristic	(n = 25)			(n = 27)		
Gender, male	–	–	65.4	–	–	81.5
Age	54.0	3.9	–	54.0	5.6	–

### Outcomes

#### Time Until Full RTW

**Fig. 2:**
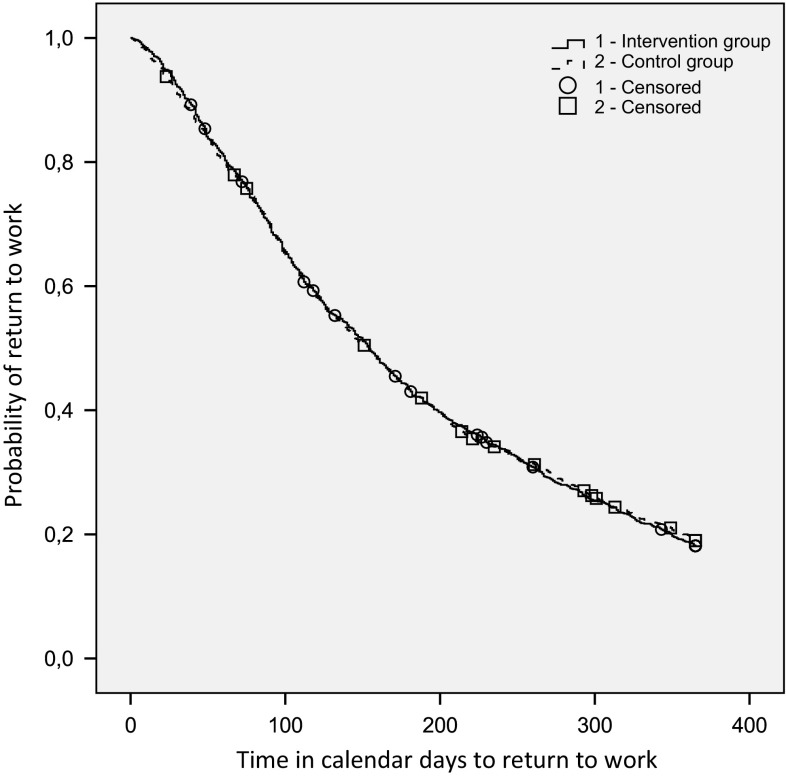
Kaplan–Meier curve time to full return to work

The differences in time to full RTW between the two groups are illustrated with the Kaplan–Meier survival curve, see Fig. 2. The number of workers who established full RTW, and the mean and median time until full RTW, were comparable between both groups (see Table 2). The hazard ratio of the intervention compared to the control group was 0.96 (95% CI 0.81–1.15), indicating that workers in the intervention group and in the control group had the same likelihood of full RTW during the follow-up period. Adjustments for baseline characteristics (age, gender and number of contract working hours per week) yielded a comparable hazard ratio 0.97 (95% CI 0.82–1.16). As some workers had been treated by several different OPs (e.g. during holidays, or reorganizations), an additional analysis on workers guided by only one OP (n = 2796) was done, which showed a comparable hazard ratio of 0.99 (95% CI 0.81–1.20).

**Table 2:** Return to work outcomes per group

	Intervention group				Control group				HR	95% CI
	(n = 1429)				(n = 1799)					
	%	Median	Mean	SD	%	Median	Mean	SD		
Full RTW^a^ after 12 months follow-up	81	–	–	–	81	–	–	–	–	–
Full RTW^a^ total follow-up	91	–	–	–	89	–	–	–	–	–
Days to full RTW^a^	–	–	212	158	–	–	214	182	–	–
Days to full RTW^a^	–	154	–	–	–	154	–	–	0.96	0.81–1.15
First RTW^a^ after 12 months follow-up	89	–	–	–	87	–	–	–	–	–
First RTW^a^ total follow-up	93	–	–	–	91	–	–	–	–	–
Days to first RTW^a^	–	–	151	173	–	–	158	185	–	–
Days to first RTW^a^	–	91	–	–	–	93	–	–	0.96	0.80–1.15

^a^ *RTW* return to work

#### Time Until First RTW

The mean and median time to first RTW and the number of workers who established their first RTW within 1 year after the start of the sickness absence were comparable in both groups (see Table 2). The hazard ratio of the intervention compared to the control group was 0.96 (95% CI 0.80–1.15), indicating that workers in the intervention group and in the control group had the same likelihood of having a first RTW during the follow-up period. Adjustments for baseline characteristics (age, gender, and number of contract working hours per week) yielded a comparable hazard ratio 0.96 (95% CI 0.80–1.15). An additional analysis on workers guided by only one OP (n = 2796) showed a comparable hazard ratio 1.01 (95% CI 0.84–1.22).

#### Total Hours of Sickness Absence

The estimated mean for total hours of sickness absence was 478 (95% CI 425–530) in the intervention group and 483 (95% CI 436–531) in the control group (−5.51 (95% CI −76 to 65), *p* = 0.88).

## Discussion

Although the intervention had shown to be effective in improving OPs’ self-reported guideline adherence [17], the present study showed that it was not effective in reducing the sickness absence duration in workers with CMD. Moreover, no differences were found for the total hours of sickness absence due to CMD in the 12 months after the start of the sick leave.

There are various possible explanations. A first option is that, notwithstanding that we know from the feasibility study that the intervention was completed as planned and that the perceived guideline adherence improved [17], the factual provided care to the workers did not improve. A previous study has shown that self-reported guideline adherence is not an accurate measure of guideline adherence [27], and therefore OPs may have overestimated their own behavior. In addition to the current study, the effect of the intervention on OPs’ actual adherence was evaluated, and some improvement of OPs’ guideline adherence was found (Joosen et al., submitted). Hence, it is possible that this small improvement did not lead to optimal guideline-based care by OPs.

Second, even if OPs’ knowledge, attitude or even factual behavior did improve, this might not have led to real improvement because of remaining conditional external barriers [17]. During and after the training OPs perceived many conditional external barriers for guideline use, such as lack of time and lack of facilities to actually follow the guideline. For example, this was due to financial contracts between employers and OHS limiting the number of contacts between OP and worker, and the conflicting policy of and lack of collaboration with for example employer and other (mental) health care providers [28]. Besides, in general OPs experience a high increase of the OPs’ workloads [29]. Although the intervention enhanced OPs’ knowledge, attitudes, perceived skills, and perceived guideline adherence, it is possible that the remaining conditional external barriers, such as very limited time and possibilities to see the worker, prevented these positive effects to lead to an effective practice.

A third possible explanation is that the guideline in its present form is not effective, and workers need a different kind of guidance in order to return to their work earlier. However, a retrospective study [7] and a process evaluation of a randomized trial [10] showed several elements of the guideline to be significantly related to an earlier RTW [7]. Furthermore, the guideline recommendations are based on and supported by evidence from a variety of studies [6, 30, 31], such as regarding the fact that relapse prevention is important [32], which makes it unlikely that the guideline is not effective.

In combination with and in addition to the former point a fourth possible explanation is that the contrast between the intervention and the control groups may have been insufficient. A RCT does not reveal absolute effectiveness, but effectiveness relative to the control group. In our study all OPs were not only supposed to work according to the Dutch occupational health guideline, but also, since the introduction of this guideline, the idea has become common among Dutch OPs that earlier work resumption can contribute to recovery, which is a key recommendation in the guideline. This was not yet the case in the late 1990s when the study of van der Klink et al. [6] found their intervention to be effective. The deficiency to find an effect might thus reflect a lack of contrast relative to care as usual that changed considerably in the past 15 years in the Netherlands, rather than an absolute lack of efficiency of the guideline.

A combination of these factors may also be an explanation for the fact that, in spite of many years of research, it seems difficult to develop interventions that are successful in reducing the sickness absence duration in workers with mental health problems. The findings of this study add to a series of RCTs in which interventions were developed to reduce sickness absence duration in workers with CMD [33–36]. Most of these studies were conducted in The Netherlands, and the interventions were not effective in reducing workers’ sickness absence duration [34–36]. In some previous studies implementation problems interfered with the developed interventions and as such also with the findings on the interventions’ effectiveness [34, 36, 37]. Very few previous studies have found a positive effect on sickness absence duration [6, 30, 33]. In all these effective studies OPs could spend time on guidance and contacts with the company. The intervention in one of these studies focused both on occupational professionals and on workers [33], which contrasts with most other interventions that primarily focus on professionals. Moreover, remarkably, in most recent studies, the time to workers’ RTW was long-lasting [34–36], which might reflect the growing experienced work pressure and demands by Dutch workers [38].

The present study has several strengths and limitations that need to be discussed. A strength of this study was the cluster RCT design, which limited the possibility of contamination between the intervention group and the control group. To prevent selection bias, the workers were selected from the registration system of the OHS after their first consultations with participating OPs. Another strength was the large sample size, of 3379 workers, which made it more likely to have reliable outcomes. The data on these workers were extracted from the OHS registration system to prevent recall bias which could occur in workers with CMD. The drawback of using the OHS registration system for data extraction, was the limited number of baseline characteristics available, such as specific diagnosis and severity, information about the work context, and treatment by other (mental) health care professionals. Preferably, these would have been used as possible confounders or possible effect modifiers in the analyses, providing better explanations for the findings. However, due to the randomized controlled trial design, it expected that these aspects were similar in both groups. Another limitation is the lack of the assessment of OPs’ actual guideline adherence that might have given more information to explain the found results. More comprehensive outcomes were collected for the smaller sample of 128 workers and the results of these evaluations will be published separately.

Overall, the intervention developed to enhance OPs’ guideline adherence in this study did not reduce the sickness absence duration in workers with CMD. Several possible explanations were given for this lack of effectiveness. Future research should further explore the implementation process and the effect of the implementation strategy on the provided occupational health care, preferably in a mixed methods design. If conditional extern barriers for using the guideline actually impede optimal guideline-based care, than future research should also focus on the organization of occupational health care beside the one-sided focus on interventions for occupational professionals. Furthermore, recently a positive effect on RTW was found for a decision aid for OPs combined with an e-health module for workers [33]. Possibly guideline-based care can be improved by providing such tools for occupational professionals and workers. In general, recent studies have shown CMD workers’ long-lasting sickness absence duration whereby mental health problems remain a large problem for society. Future research and practice should continue the search on how to solve this problem.

## Abbreviations

CMD: Common mental disorders
NVAB: Netherlands Society of Occupational Medicine
RTW: Return to work
OP: Occupational physician
RCT: Randomized controlled trial
OHS: Occupational health service
ICD-10: International Classification of Diseases version 10
